# Fear-Potentiated Behaviour Is Modulated by Central Amygdala Angiotensin II AT_1_ Receptors Stimulation

**DOI:** 10.1155/2014/183248

**Published:** 2014-06-09

**Authors:** Maria de los Angeles Marinzalda, Pablo A. Pérez, Pascual A. Gargiulo, Brenda S. Casarsa, Claudia Bregonzio, Gustavo Baiardi

**Affiliations:** ^1^Laboratory of Neuropharmacology, Institute of Biological and Technological Research (IIBYT-CONICET), National University of Córdoba, Faculty of Chemical Sciences, Catholic University of Córdoba, 5017 Córdoba, Argentina; ^2^Laboratory of Neurosciences and Experimental Psychology, IMBECU-CONICET, Department of Pathology, Faculty of Medical Sciences, National University of Cuyo, 5500 Mendoza, Argentina; ^3^Institute of Experimental Pharmacology (IFEC-CONICET), Department of Pharmacology, Faculty of Chemical Sciences, National University of Córdoba, 5000 Córdoba, Argentina

## Abstract

Central nucleus of the amygdala (CeA) is one of the most important regulatory centres for the emotional processes. Among the different neurotransmitter systems present in this nucleus, AT_1_ receptors have been also found, but their role in the generation and modulation of emotions is not fully understood. The present work evaluated the effect of intra-amygdalar injection of losartan (AT_1_ receptor antagonist) and angiotensin II (Ang II) in the anxiety state induced by fear-potentiated plus maze in male Wistar rats. Fear in the elevated plus maze can be potentiated by prior inescapable footshock stress. The decrease in the time spent in the open arms induced by the inescapable footshock was totally prevented by losartan (4 pmol) administration in CeA. It was also found that Ang II (48 fmol) administration decreased the time spent in the open arms in animals with or without previous footshock exposure. The locomotor activity and grooming behaviour were also evaluated. The results obtained from the different parameters analyzed allowed us to conclude that the Ang II AT_1_ receptors in CeA are involved in the anxiety state induced by stress in the fear-potentiated plus-maze behaviour.

## 1. Introduction

All the components of the renin angiotensin system, including the receptors, have been found in brain tissue, indicating a role as a hormone or neuromodulator in the central nervous system [1, 2]. Angiotensin II (Ang II) exerts its principal known actions at the AT_1_ receptor. AT_1_ receptors are located in brain areas related to the control of neuroendocrine functions and the autonomic regulation of limbic and cardiovascular systems. The role of brain Ang II is complex and is related to control of the autonomic and hormonal system and sensorial and cognitive processes including regulation of cerebral blood flow [2, 3].

There is a large body of evidence, at pharmacological, neuroanatomical, and physiological level, supporting a key role for Ang II in the stress response, including regulation of the sympathetic and neuroendocrine systems. However, the results obtained using AT_1_ blockers as a pretreatment in animals exposed to different stress conditions, such as isolation, cold restraint, or novelty, suggest that their actions may not be limited to the hypothalamus-pituitary-adrenal axis only. In addition, pretreatment with candesartan, an AT_1_ blocker, prevents isolation-induced decrease in CRH_1_ receptors and the GABA_A_ complex in the brain cortex [4]. When the animals were tested in the plus maze they exhibited an increase in the parameters associated with anxiogenic effects. All these together strongly suggest a role for AT_1_ receptors not only in autonomic and hormonal but also in behavioural response to stress [4, 5].

The central nucleus of the amygdala (CeA) plays a critical role in integrating sympathetic and behavioural responses to stress [6–8]. The CeA is necessary for learning increased alertness to conditioned fear [9]. There are extensive and often reciprocal projections between the CeA and nuclei in the hypothalamus and medulla that regulate autonomic and cardiac functions [10, 11]. These observations underscore the importance of the CeA in modulating the hemodynamic and behavioural responses to stress. The CeA contains GABA receptors [12] that have been shown to inhibit hemodynamic and behavioural responses to stress [7]. The CeA also contains Ang II, angiotensin converting enzyme, and AT_1_ receptors [13] and it has been shown that Ang II is a key neurotransmitter in the CeA involved in regulation of sympathetic and hemodynamic responses to stress [8].

The accumulated evidences point to the central nucleus of the amygdala as the structure by which the information leaves the amygdala in the fear conditioning circuitry; lesions in this nucleus prevent both simple and differential conditioning in the fear response.

The purpose of this study was to determine the role of CeA Ang II and AT_1_ receptors in the potential fear induced by previous footshock stress measured as a decreased percentage of time spent on open arm exploration in the elevated plus maze. The fear-potentiated plus maze test may be a valuable tool in the search for novel anxiolytics and in the study of the neurobiology of fear potentiation, fear conditioning, and fear generalization [14].

## 2. Materials and Methods

### 2.1. Animals

Wistar male rats, aged 90 days and weighing 250–300 g, were used. They were maintained under controlled conditions of temperature (21–23°C), humidity (20–25%), and light cycle of 12L : 12D (7:00–19:00 h). Standard rat food and water were freely available. All procedures were handled in accordance with the guidelines set by European Community Council (Directive 86/609/EEC), as approved by the Animal Care and Use Committee of the National University of Córdoba, Argentina.

### 2.2. Drugs

Losartan or Ang II (Sigma-Aldrich) dissolved in 1 *μ*L of saline solution, immediately before the injection, was used.

### 2.3. Surgery

Animals were anesthetized with ether and stereotaxically implanted with bilateral stainless steel cannula into the CeA. Coordinates for cannulae implantation were anterocaudal: −2.4; lateral: ±4.0; vertical: −7.2 mm, according to the Atlas of Paxinos [15]. The cannula consisted of an outer guiding cannula stainless steel tubing (23-gauge, 15 mm in length) and an inner removable stylet (30-gauge, 15 mm in length) to prevent the obstruction. After surgery, rats were housed individually and maintained undisturbed in recovery for a week.

### 2.4. Experimental Design for the Fear-Potentiated Behaviour Model

In order to study the effects of AT_1_ receptor blockade and Ang II on fear-potentiated plus-maze behaviour [14] two groups of animals were used. The first group (control) of rats received no shocks. The second group of rats (fear-potentiated) received three footshocks 0.3 mA during 3 s separated by 30 s interval in a conditioning compartment on the first day. On the second day, the rats were exposed to their own training compartment, but this time the rats received no shocks (contextual conditioning). Directly thereafter, the animals received intra-amygdalar injections before being exposed to plus maze test. The following 6 groups: control saline, control Ang II; control losartan, fear-potentiated saline, fear-potentiated Ang II, and fear-potentiated losartan were tested.

### 2.5. Intra-Amygdalar Microinjection of Drugs

The rats were gently wrapped in a cloth and manually restrained and injected bilaterally into the CeA using a 30 gauge stainless steel injection needles attached to a 10 *μ*L microsyringe (Hamilton Company, USA) by polyethylene tubing, introduced into the guide cannula (0.5 mm below the tip of the guide cannula). The animals received saline (artificial cerebrospinal fluid), losartan 4 pmol (specific AT_1_ receptor antagonist), or Ang II 48 fmol and 15 min later they were tested in the plus maze. The injection solutions were administered in a volume of 0.5 *μ*L in each side (gradually injected over the period of 1 min) into the CeA. The injection needles were left in place for additional 20 s to allow diffusion. The doses were selected on the basis of preliminary reports [16].

### 2.6. Apparatus and Behavioural Test

The plus maze was made of wood and consisted of two open arms (50 × 10 cm) and two enclosed arms (50 × 10 × 40 cm); it was elevated 50 cm above the ground. A video camera was mounted vertically over the plus maze and the rat's behaviour was recorded and digitalized on a computer located in an adjacent room. The testing room was quiet and dimly lit. Fifteen minutes after injection, each rat was placed in the central square of the plus maze, facing the closed arm, and was allowed 5 min to freely explore the maze. The time spent on the open arms, the open arm entries, extreme arrivals, the number of closed arm entries, and total distance was obtained using Tracker 4.62 software (open source physics. 2012). Grooming behaviour was considered an adaptation to stressful situations [17]. It includes nonambulatory stereotyped movements such as vibrating movements of forelegs, washing of forelegs and head, and cleansing of hind legs, tail, body, genitals, and scratching. The rats in video files were observed and scored as described [18]. All the sessions took place between 10 am and 1 pm.

### 2.7. Statistical Analysis

The Kolmogorov Smirnov test was used to ascertain Gaussian distribution of data. The data were analyzed using two-way ANOVA with saline, Los, and Ang II as drug factor and fear-potentiated and control as condition factor. If an interaction (condition × drug) and/or main effect were observed, pairwise comparisons were made using Bonferroni* post hoc* test. In all cases, a *P* < 0.05 (two tailed) was considered significant. The results are reported as the mean ± SEM at *n* = 10–13 for each group.

## 3. Results

### 3.1. Histology

**Figure 1:**
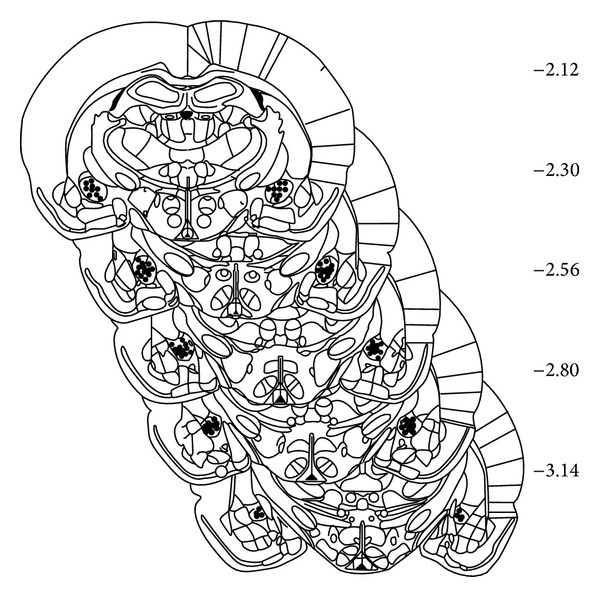
Histological cannulae placement of rats used in the plus maze test. Schematic representation of histological findings in frontal brain sections showing the location of the injection site. The values at the right represent the distance in respect of bregma (modified from Paxinos, 1997). ● Injection placement.

When the testing was completed, the rats were sacrificed by decapitation and the brains were removed from the skull and fixed in 20% formalin solution. The brains were mounted and frozen in a cryotome and cut into 40 *μ*m sections. The block face was examined with 40x magnifying lens and the sections containing the injection sites were analyzed. Microscopic inspection of these sections served to ascertain the location of the cannulae tips. The locations were transferred to standard sections taken from a brain atlas [15]. Seven of the 72 operated rats were excluded from data analysis. The cannulae were not correctly positioned in the CeA. Only the data for those rats which had correct CeA cannulae placements was reported (Figure 1).

### 3.2. Closed Arms Entries and Total Distance

**Figure 2:**
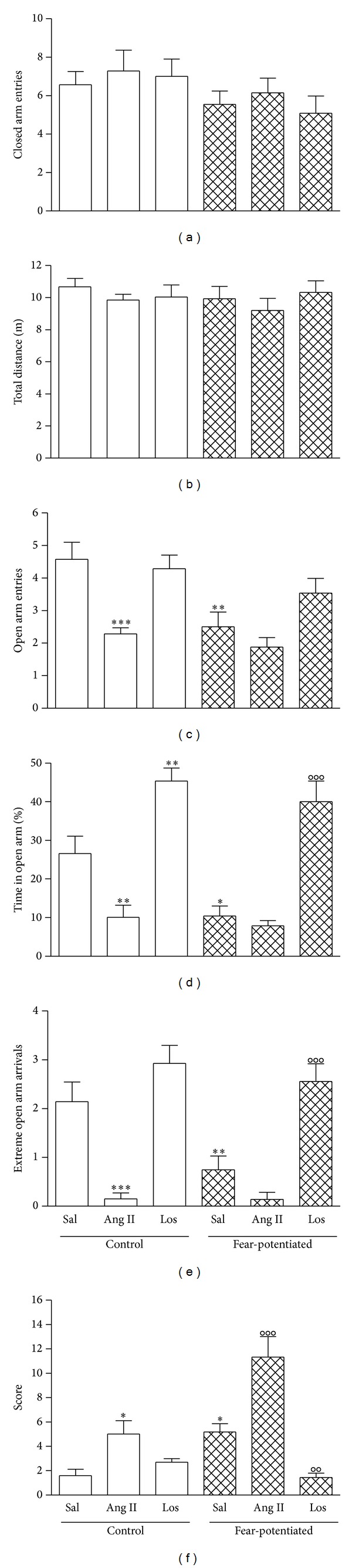
Number of closed arm entries (a), total distance (b), open arm entries (c), time spent in the open arms (d), number of extreme open arm arrivals (e), and grooming behaviour (f). The data are expressed as the mean ± SEM. Saline (Sal), angiotensin II (Ang II), and losartan (Los). *P < 0.05, **P < 0.01, and ***P < 0.001 versus control saline group. ^oo^ *P* < 0.01 and ^ooo^ *P* < 0.001 versus fear-potentiated saline group.

The number of closed arms entries and the total distance are index of locomotor activity developed by each animal during the test [19]. The statistical evaluation by two-way ANOVA for the number of closed arm entries showed no significant effect of condition (fear-potentiated, control) *F*(1,59) = 3.78, *P* = 0.057, drug (saline, Los, and Ang II) *F*(2,59) = 0.4, *P* = 0.67, and interaction (drug × condition) *F*(2, 59) = 0.16, *P* = 0.85 (Figure 2(a)). Also, the total distance showed no significant effect of condition *F*(1, 59) = 0.42, *P* = 0.52, effect of drug *F*(2,59) = 0.73, *P* = 0.48, and interaction between both factors *F*(2, 59) = 0.34, *P* = 0.71 (Figure 2(b)).

### 3.3. Time Spent in Open Arms and Open Arm Entries

The number of entries and the percentage of time spent in the open arms were evaluated as anxiety index. This is a spatial-time behaviour validated in rats exposed to different experimental conditions [20, 21].

The open arm entries showed a significant effect of condition *F*(1,59) = 9.98, *P* < 0.003. Subsequent pairwise comparisons by Bonferroni* post hoc *test indicated that there was a decrease in the fear-potentiated with respect to control condition in saline injected animals (4.57 ± 0.53 versus 2.5 ± 0.45, *t* = 3.54  *P* < 0.01).

Significant effect of drug *F*(2,59) = 10.73, *P* < 0.0001, was found. Ang II injection decreased the number of open arm entries in control condition (4.57 ± 0.53 versus  2.3 ± 0.18, *t* = 3.74  *P* < 0.001), but not in fear-potentiated condition (2.5 ± 0.45 versus 1.88 ± 0.3, *t* = 1.07  *P* > 0.05) compared to saline injected animals. There was no effect of losartan in both control and fear-potentiated conditions compared to saline injected group (4.3 ± 0.4 versus 4.7 ± 0.5  *t* = 0.47  *P* > 0.05 and 3.5 ± 0.4 versus 2.5 ± 0.4  *t* = 1.9  *P* > 0.05, resp.). Nonsignificant effect of interaction (drug × condition) was found, *F*(2,59) = 2.21, *P* = 0.12, (Figure 2(c)).

The time spent in open arms showed a significant effect of condition (control, fear-potentiated) *F*(1,59) = 6.43, *P* < 0.02. Bonferroni* post hoc* test analysis showed a significant decrease in fear-potentiated with respect to control condition in saline injected animals (26.6 ± 4.5 versus 10.38 ± 2.6, *t* = 3.02, *P* < 0.05); these results are in accordance with previous findings using this animal model [14]. Drug effect (saline, Los, and Ang II) was highly significant *F*(2,59) = 41.19, *P* < 0.0001. Ang II injection decreased the time spent in the open arms with respect to saline treated animals in control condition (26.6 ± 4.5 versus 10.1 ± 3.1, *t* = 2.9  *P* < 0.01); this response was not observed in fear-potentiated condition (10.4 ± 2.6 versus 7.8 ± 1.4  *t* = 0.47  *P* > 0.05). Losartan injection in control and fear-potentiated condition induced a marked increase in this parameter with respect to saline injected animals (26.6 ± 4.5 versus 45.4 ± 3.4, *t* = 3.35  *P* < 0.01 and 10.37 ± 2.6 versus 40.0 ± 5.4  *t* = 5.9  *P* < 0.001, resp.) showing a complete reversion of the anxiogenic effect induced by fear-potentiated condition. No interaction effect was found, *F*(2,59) = 1.8, *P* < 0.17, (Figure 2(d)).

### 3.4. Extreme Arrivals

The arrival to the extreme portion of the open arms was considered another anxiety index [21, 22]. Two-way ANOVA showed a significant effect of condition (fear-potentiated, control) *F*(1,59) = 5.42, *P* < 0.05. This behaviour was significantly decreased by fear-potentiated condition (2.14 ± 0.4 versus 0.75 ± 0.3, *t* = 3.2  *P* < 0.01) and it showed a very significant effect of drug *F*(2,59) = 35.04, *P* < 0.0001. Ang II injection decreased this parameter in control conditions compared to saline injected animals (2.14 ± 0.40 versus 0.15 ± 0.12  *t* = 4.4, *P* < 0.001) but none in fear-potentiated condition (0.14 ± 0.15 versus 0.75 ± 0.3, *t* = 1.4  *P* > 0.05). Losartan administration clearly reversed the decrease induced by fear-potentiated condition in saline injected animals (0.75 ± 0.3 versus 2.56 ± 0.36  *t* = 4.5, *P* < 0.001). No interaction effect was found, *F*(2,59) = 2.6, *P* = 0.08 (Figure 2(e)).

### 3.5. Grooming Behaviour

The grooming is behaviour without spatiotemporal activity in the maze. It is sensitive to stress induced by novelty environment [18].

Statistical analysis of data revealed a significant effect of condition (fear-potentiated, control) *F*(1,59) = 16.34  *P* < 0.001. There was a significant increase in fear-potentiated saline group with respect to control saline (1.59 ± 0.53 versus 5.18 ± 0.68, *t* = 2.93  *P* < 0.05), indicating that exposure to fear-potentiated condition increased this behaviour. Drug effect (saline, Los, and Ang II) was highly significant *F*(2,59) = 26.95, *P* < 0.0001. Ang II injection increased the score of grooming behaviour in control condition (1.59 ± 0.53 versus 5.0 ± 1.1, *t* = 2.7  *P* < 0.05) and in fear-potentiated condition compared to saline group (5.18 ± 0.68 versus 11.33 ± 1.69, *t* = 5.0  *P* < 0.001). The stimulation by Ang II injection was higher than fear-potentiated stimulation (5.18 ± 0.68 versus 11.33 ± 1.69, *t* = 5.0  *P* < 0.001). Losartan decreased this behaviour only in fear-potentiated condition compared to saline injected animals (5.18 ± 0.68 versus 1.43 ± 0.37, *t* = 3.28  *P* < 0.01) showing that fear-potentiated condition can induce grooming behaviour by stimulation of CeA AT_1_ receptors. In this case, there is a significant drug × condition interaction effect *F*(2,59) = 9.67, *P* < 0.001, indicating that the drug effect depends on condition (Figure 2(f)).

## 4. Discussion

The main finding of this work is that Ang II AT_1_ receptors in CeA are involved in the fear conditioning process expressed in the fear-potentiated plus-maze behaviour. The model of fear-potentiated plus-maze behaviour chosen for the present work gives a valuable measure in the understanding of neural mechanisms involved in the anxiety state and in the search for novel anxiolytics, in contrast to the normal elevated plus maze, which measures innate fear of open spaces; fear-potentiated plus-maze behaviour reflects an enhanced anxiety state [14]. The neural mechanisms involved in fear-potentiated plus-maze behaviour (state anxiety) compared to spontaneous plus-maze behaviour (trait anxiety) are quite different because in anxiety state a cognitive appraisal of threat is a prerequisite for the experience of this type of emotion, whereas, in trait anxiety, the existence of stable individual differences is characteristic. Moreover, it is known that fear conditioning processes may contribute to such disorders as phobia, excessive fear, anxiety, posttraumatic stress, and panic [23, 24]. For these reasons, studying fear-potentiated plus-maze behavior is of interest. In fear conditioning, both hippocampus and amygdala play important roles. Since Ang II AT_1_ receptors are present in the brain and there is a growing body of evidences supporting a key role for these receptors in the stress response at different brain levels and in the amygdala it was considered important to study the possible role of this system in this behavioural model.

In our experiments, it was corroborated that the fear-potentiated condition decreased the time spent in the open arms in the elevated plus maze. The losartan administration in CeA totally prevented this response, showing that the AT_1_ receptor blockade induced an anxiolytic effect. Meanwhile, losartan did not affect the number of open arms entries in control and fear-potentiated conditions compared to saline injected group. The closed arms entries and the total distance were not affected in any of the experimental conditions analyzed. This last one strongly indicates that the change found in the time spent in the open arms is only a reflection of anxiety because the locomotor activity was not affected.

Emotional aspects of Ang II activity have been raised by several authors. Anxiolytic properties of the AT_1_ receptor antagonist losartan described suggested anxiogenic potency of the AT_1_ stimulation [25]. This was supported by studies showing reversal of anxiogenic action of i.c.v. Ang II by an equimolar (low) dose (2 nmol) of i.c.v. losartan [26]. Moreover, anxiogenic profile of transgenic (mREN2) 27 rats characterized by increased level of brain angiotensin and fulminant hypertension [27] points to the increased emotionality caused by Ang II.

Ang II and AT_1_ receptors exist in the amygdala and, in particular, in the CeA [13]. Microinjection of Ang II in the amygdala of the rat increases the discharge rate of amygdalar neurons and the increase can be blocked by AT_1_ receptor antagonists [28]. Although studies of the specific role of Ang II in the amygdala are limited, it has been shown that Ang II microinjection into the CeA reduced sexual behaviour [29] and the blockade of AT_1_ or AT_2_ receptors in the CeA prevented the effect of stress and the effect of Ang II microinjection into this nucleus on sexual receptivity [30]. Moreover, the CeA has been implicated in anxiety, meanwhile, the basolateral amygdala in the stress response and memory [16]. The above evidences agree with our present findings that show an anxiogenic effect of Ang II in CeA when tested in the plus maze. The Ang II effects could be mediated by its action on AT_1_ receptor based on the evidences pointed above [13, 28, 29] and the results obtained with the AT_1_ receptor antagonist used in the present work. This would explain the decrease in the parameter of open arm entries, time spent in open arms, and extreme arrivals induced by Ang II injection in control conditions interpreted as an anxiogenic effect. Interestingly, the Ang II administration in fear-potentiated condition did not modify the time spent in open arms or extreme arrivals. This could be because the fear-potentiated condition induced a greater stimulation of AT_1_ receptor reaching the maximum response for this behaviour and did not invalidate the results obtained with Ang II in control conditions because each behaviour could have different maximum values.

It has been shown that AT_1_ receptor antagonists reduce stress responses and anxiety in rodents preventing sympathoadrenal response to stress and its gastric consequences and confirming the role of Ang II as a stress hormone [31–33]. Peripheral administration of losartan attenuated motor hyperactivity and anxiogenic behaviour in hypertensive rats and induced anxiolysis in normotensive rats showing a behavioural profile very similar to diazepam, as observed in elevated plus maze and social interaction tests [34]. In the same way, intracerebroventricular administered valsartan, another AT_1_ antagonist, has shown anxiolytic-like effects in the plus maze test [35]. Blockade of the AT_1_ receptors or angiotensin converting enzyme inhibition decreases hypothalamus-pituitary-adrenal axis reactivity independently from the blood pressure decrease [36].

Our data indicate that the parameters, time spent in the open arms, and extreme arrivals are the most sensitive markers to stress because the footshock stress and reexposure to stress context significantly decrease them. In this sense, our findings, with respect to the degree of decrease in the time spent in the open arms induced by context reexposure, agree with the findings of Korte and De Boer [14].

Since losartan microinjection in CeA totally reversed the anxiogenic effect induced by fear-potentiated it could be suggested that the Ang II AT_1_ receptors in this brain area are involved in the generation of the anxiety state.

Another parameter analyzed was the grooming behaviour, characterized as a nonambulatory stereotyped movement and described as a response associated with the restoration of homeostasis in a stress situation. In this way, the animals under fear-potentiated condition would spend more time in grooming than the animals in control condition [18]. This could explain our findings with Ang II microinjected in CeA that significantly increased the grooming behaviour score. Losartan microinjected in CeA blunted the increase in the grooming behaviour induced by fear-potentiated condition, suggesting that the AT_1_ receptors in CeA are involved in the stress response associated with this behaviour. This is in accordance with our previous findings, showing that intra-amygdalar AT_1_ receptor blockade has an anxiolytic effect when tested in the plus maze under basal conditions or after a previous restraint stress [16]. Interestingly, other laboratories found that losartan enhances the extinction of fear memory and they also found a decrease of AT_1_ receptors in amygdala of losartan treated mice [24]. These findings support the view that the AT_1_ receptors are involved in the aversive memory giving a role for Ang II in fear-related neurobiological processes. The results of the present study where the anxiety state is induced by fear, involving an emotional learning blunted by intra-amygdalar losartan administration, are according to the findings described above. Based on these last evidences, it is possible to suggest that the Ang II AT_1_ receptors activation in the amygdala could be playing a central role in the emotional learning process.

The evidences point to Ang II as a peptide that facilitates the dopamine release through the AT_1_ receptors on presynaptic neurons [37–39]. It has been found that AT_1_ receptor activation in the hypothalamus and striatum produced an increase in extracellular dopamine levels [38, 40] and this effect was totally abolished by losartan [38]. The increase in dopamine levels in the striatum is generally associated with augmented locomotor activity; meanwhile in the amygdala it is related to anxiogenic behavioural responses [40]. Based on these evidences, it is possible to suggest that the AT_1_ receptor blockade would be affecting the dopamine levels in amygdala as it was described in other brain areas.

The stimulation of the hypothalamic-pituitary-adrenal-axis induced by neuronal amygdala activation, modulated by AT_1_ receptors, could induce CRH synthesis and release and stimulate noradrenergic activity in the amygdala and PVN and mediate in part the glucocorticoid release that occurs in stress conditions [8].

Considering the results obtained in the present work, together with the previous evidences, it is possible to postulate that AT_1_ receptors in CeA are involved in the generation of the anxiety state. The studies on the physiological and pathological role of brain Ang II aim to encourage the study of this system in the context of the search for new pharmacological tools in the treatment of stress-related disorders.

## References

[1] Lavoie JL, Sigmund CD (2003). Minireview: overview of the renin-angiotensin system—an endocrine and paracrine system. *Endocrinology*.

[2] Danser AHJ (2003). Local renin-angiotensin systems: the unanswered questions. *International Journal of Biochemistry and Cell Biology*.

[3] Saavedra JM (1992). Brain and pituitary angiotensin. *Endocrine Reviews*.

[4] Saavedra JM, Armando I, Bregonzio C (2006). A centrally acting, anxiolytic angiotensin II AT1 receptor antagonist prevents the isolation stress-induced decrease in cortical CRF 1 receptor and benzodiazepine binding. *Neuropsychopharmacology*.

[5] Shekhar A, Sajdyk TJ, Gehlert DR, Rainnie DG (2003). The amygdala, panic disorder, and cardiovascular responses. *Annals of the New York Academy of Sciences*.

[6] Bohus B, Koolhaas JM, Korte SM, Roozendaal B, Wiersma A (1996). Forebrain pathways and their behavioural interactions with neuroendocrine and cardiovascular function in the rat. *Clinical and Experimental Pharmacology and Physiology*.

[7] Saha S (2005). Role of the central nucleus of the amygdala in the control of blood pressure: descending pathways to medullary cardiovascular nuclei. *Clinical and Experimental Pharmacology and Physiology*.

[8] Watanabe MA, Kucenas S, Bowman TA, Ruhlman M, Knuepfer MM (2010). Angiotensin II and CRF receptors in the central nucleus of the amygdala mediate hemodynamic response variability to cocaine in conscious rats. *Brain Research*.

[9] Walker DL, Davis M (2000). Involvement of NMDA receptors within the amygdala in short- versus long-term memory for fear conditioning as assessed with fear-potentiated startle. *Behavioral Neuroscience*.

[10] Pitkänen A, Pikkarainen M, Nurminen N, Ylinen A (2000). Reciprocal connections between the amygdala and the hippocampal formation, perirhinal cortex, and postrhinal cortex in rat. *Annals of the New York Academy of Sciences*.

[11] Gray TS, Carney ME, Magnuson DJ (1989). Direct projections from the central amygdaloid nucleus to the hypothalamic paraventricular nucleus: possible role in stress-induced adrenocorticotropin release. *Neuroendocrinology*.

[12] Marowsky A, Fritschy JM, Vogt KE (2004). Functional mapping of GABAA receptor subtypes in the amygdala. *European Journal of Neuroscience*.

[13] von Bohlen und Halbach O, Albrecht D (1998). Visualization of specific angiotensin II binding sites in the rat limbic system. *Neuropeptides*.

[14] Korte SM, de Boer SF (2003). A robust animal model of state anxiety: fear-potentiated behaviour in the elevated plus-maze. *European Journal of Pharmacology*.

[15] Paxinos G (1997). *The Rat Brain in Stereotaxic Coordinates*.

[16] Llano-López LH, Caif F, García S (2012). Anxiolytic-like effect of losartan injected into amygdala of the acutely stressed rats. *Pharmacological Reports*.

[17] Nin MS, Couto-Pereira NS, Souza MF (2012). Anxiolytic effect of clonazepam in female rats: grooming microstructure and elevated plus maze tests. *European Journal of Pharmacology*.

[18] Gispen WH, Isaacson RL (1981). ACTH-induced excessive grooming in the rat. *Pharmacology and Therapeutics*.

[19] Gonzalez LE, File SE (1997). A five minute experience in the elevated plus-maze alters the state of the benzodiazepine receptor in the dorsal raphe nucleus. *Journal of Neuroscience*.

[20] Lister RG (1990). Ethologically-based animal models of anxiety disorders. *Pharmacology and Therapeutics*.

[21] Wall PM, Messier C (2001). Methodological and conceptual issues in the use of the elevated plus-maze as a psychological measurement instrument of animal anxiety-like behavior. *Neuroscience and Biobehavioral Reviews*.

[22] Fernandes C, File SE (1996). The influence of open arm ledges and maze experience in the elevated plus-maze. *Pharmacology Biochemistry and Behavior*.

[23] LeDoux JE (1995). Emotion: clues from the brain. *Annual Review of Psychology*.

[24] Marvar PJ, Goodman J, Fuchs S, Choi DC, Banerjee S, Ressler KJ (2014). Angiotensin type 1 receptor inhibition enhances the extinction of fear memory. *Biological Psychiatry*.

[25] Kaiser FC, Palmer GC, Wallace AV, Carr RD, Fraser-Rae L, Hallam C (1992). Antianxiety properties of the angiotensin II antagonist, DUP 753, in the rat using the elevated plus-maze. *NeuroReport*.

[26] Kułakowska A, Karwowska W, Wiśniewski K, Braszko JJ (1996). Losartan influences behavioural effects of angiotensin II in rats. *Pharmacological Research*.

[27] Wilson W, Voigt P, Bader M, Marsden CA, Fink H (1996). Behaviour of the transgenic (mREN2)27 rat. *Brain Research*.

[28] Albrecht D, Nitschke T, von Bohlen Und Halbach O (2000). Various effects of angiotensin II on amygdaloid neuronal activity in normotensive control and hypertensive transgenic [TGR(mREN-2)27] rats. *The FASEB Journal*.

[29] Breigeiron MK, Morris M, Lucion AB, Sanvitto GL (2002). Effects of angiotensin II microinjected into medial amygdala on male sexual behavior in rats. *Hormones and Behavior*.

[30] Cecconello AL, Raineki C, Sebben V, Lucion AB, Sanvitto GL (2010). Effect of acute stress on sexual behavior in female rats: participation of the central angiotensinergic system. *Behavioural Brain Research*.

[31] Saavedra JM, Ando H, Armando I (2005). Anti-stress and anti-anxiety effects of centrally acting angiotensin II AT1 receptor antagonists. *Regulatory Peptides*.

[32] Saavedra JM, Benicky J (2007). Brain and peripheral angiotensin II play a major role in stress. *Stress*.

[33] Bregonzio C, Armando I, Ando H, Jezova M, Baiardi G, Saavedra JM (2003). Anti-inflammatory effects of angiotensin II AT1 receptor antagonism prevent stress-induced gastric injury. *The American Journal of Physiology—Gastrointestinal and Liver Physiology*.

[34] Srinivasan J, Suresh B, Ramanathan M (2003). Differential anxiolytic effect of enalapril and losartan in normotensive and renal hypertensive rats. *Physiology and Behavior*.

[35] Braszko J (2005). Valsartan abolishes most of the memory-improving effects of intracerebroventricular angiotensin II in rats. *Clinical and Experimental Hypertension*.

[36] Raasch W, Wittmershaus C, Dendorfer A (2006). Angiotensin II inhibition reduces stress sensitivity of hypothalamo-pituitary-adrenal axis in spontaneously hypertensive rats. *Endocrinology*.

[37] Brown DC, Steward LJ, Ge J, Barnes NM (1996). Ability of angiotensin II to modulate striatal dopamine release via the AT1 receptor in vitro and in vivo. *The British Journal of Pharmacology*.

[38] Raghavendra V, Chopra K, Kulkarni SK (1998). Modulation of motor functions involving the dopaminergic system by AT1 receptor antagonist, losartan. *Neuropeptides*.

[39] Georgiev V, Gyorgy L, Getova D, Markovska V (1985). Some central effects of angiotensin II. Interactions with dopaminergic transmission. *Acta Physiologica et Pharmacologica Bulgarica*.

[40] Jenkins TA, Allen AM, Chai SY, MacGregor DP, Paxinos G, Mendelsohn FAO (1996). Interactions of angiotensin II with central dopamine. *Advances in Experimental Medicine and Biology*.

